# The impact of a PERMA model-based positive psychology intervention on fear of recurrence of inflammatory bowel disease: a randomized controlled trial

**DOI:** 10.3389/fpsyg.2025.1611899

**Published:** 2025-09-11

**Authors:** Yuanyuan Qian, Linlin Ma, Jianru Hao, Lu Zhang, Ying Liu, Yongping Xu, Lifen Dai, Yanfang Luo, Zhenzhen Su

**Affiliations:** ^1^Department of Gastroenterology, Affiliated Hospital of Jiangnan University, Wuxi, China; ^2^Wuxi School of Medicine, Jiangnan University, Wuxi, China

**Keywords:** inflammatory bowel disease, PERMA model, fear of recurrence, subjective well-being, resilience, quality of life

## Abstract

**Objective:**

This study aimed to investigate the effects of a positive psychological intervention based on the PERMA model on fear of recurrence, subjective well-being, psychological resilience, and quality of life in patients with inflammatory bowel disease (IBD).

**Methods:**

This study was conducted as a single-blind, two-arm randomized controlled trial at Jiangnan University Affiliated Hospital from May to July 2024. A total of 93 hospitalized patients experiencing fear of recurrence related to IBD were randomly assigned to either the intervention group (*n* = 47) or the control group (*n* = 46). Patients in the intervention group received positive psychological intervention based on PERMA model, while patients in the control group received standard nursing. The levels of fear of recurrence, subjective well-being, resilience, and quality of life were assessed at baseline (T0), day of discharge (T1), 2 weeks post-discharge (T2), 4 weeks post-discharge (T3), and 8 weeks post-discharge (T4). Data were analyzed using independent sample *t*-tests, chi-square tests, and generalized estimating equations (GEE).

**Results:**

Resilience and quality of life scores at T2 were significantly higher in the intervention group than in the control group. As the duration of the intervention increased, there was a significant decrease in the level of fear of recurrence (T3: *U* = −1.978, *p* = 0.048; T4: *U* = −2.116, *p* = 0.034), alongside improvements in subjective well-being (T3: *t* = 2.731, *p* = 0.008; T4: *t* = 3.490, *p* < 0.001), psychological resilience (T3: *t* = 4.824, *p* < 0.001; T4: *t* = 5.699, *p* < 0.001), and quality of life (T3: *U* = −2.576, *p* = 0.010; T4: *U* = −2.746, *p* = 0.006), all of which were statistically significant. Furthermore, a significant group-related shift was noted in psychological resilience (*χ*^2^ = 14.353, *p* < 0.001). Notably, the effects of time and interaction on fear of recurrence, subjective well-being, psychological resilience, and quality of life were statistically significant (all *p* < 0.05).

**Conclusion:**

Positive psychological interventions based on the PERMA model significantly reduced fear of recurrence in IBD patients, and positively affected their subjective well-being, psychological resilience, and quality of life.

**Clinical trial registration:**

https://www.chictr.org.cn/showproj.html?proj=230313 ChiCTR2400085278.

## 1 Introduction

Inflammatory bowel disease (IBD), which is characterized by an imbalance in intestinal immune function, includes a group of disorders such as ulcerative colitis and Crohn’s disease (Hodson, 2016). However, due to changes in lifestyle and dietary patterns, China has been reported to have the highest incidence of IBD in Asia, amounting to approximately 3.44 per 100,000 individuals (Ng et al., 2017; Park and Cheon, 2021). Specifically, the primary symptoms of IBD, such as abdominal pain, diarrhea, and bloody stool, have been found to be prone to relapse, posing a persistent challenge (Kane et al., 2008). Clearly, these symptoms have been shown to significantly diminish the quality of life for affected individuals and contribute to a substantial disease burden (Calviño-Suárez et al., 2021). Currently, no accepted cure for IBD has been established (Le Berre et al., 2023), and the disease is commonly observed to recur (Jangi et al., 2021). Similarly, the global recurrence rate for ulcerative colitis (UC) has been documented at 73%, with a cumulative risk of recurrence at 1 year of 11.5% among patients receiving continuous endoscopic therapy (Rivière et al., 2021). Moreover, it has been observed that approximately 90% of patients with Crohn’s disease (CD) experience a relapse, with 70% experiencing a rapid decline within the first ten years following diagnosis, and virtually all surgical patients are at high risk of relapse (Valibouze et al., 2021). Therefore, this increased need for care places a significant burden on family life and places considerable psychological stress on the patient.

Various patients have expressed concerns regarding the adverse consequences of recurrent inflammatory bowel disease (IBD), which commonly leads to a pervasive fear of recurrence (von Wietersheim et al., 1992). However, this fear relates to anxiety surrounding the potential return or progression of an existing condition (Bisgaard et al., 2022). Similarly, the bidirectional association between IBD and anxiety or depression has been recognized as clinically significant. While this is a typical psychological response to an emergency event, excessive fear of recurrence can adversely affect a patient’s well-being. Moreover, studies involving colorectal cancer survivors have shown that fear of recurrence is periodically associated with symptoms of depression and anxiety (Custers et al., 2016). Additionally, prolonged fear of relapse has been found to result in sustained emotional distress in patients and an increased risk of depression and anxiety (Simard et al., 2013). Notably, A recent study involving cancer patients demonstrated that the impact of fear of recurrence (FoR) on quality of life (QOL) is substantial, even surpassing the effect of anxiety on QOL (Kim et al., 2012). Therefore, given these findings, fear of recurrence is emerging as a critical clinical and psychological issue among patients with inflammatory bowel disease (IBD), warranting thorough and focused attention in both clinical practice and research.

Empirical research has demonstrated that positive psychological interventions can effectively mitigate the fear of cancer recurrence among patients, thereby enhancing their overall mental health and quality of life (Lyu et al., 2022). Specifically, the field of positive psychology focuses on the identification and cultivation of patients’ positive psychological attributes, the mobilization of positive emotions, and the modulation of their sense of well-being (Duckworth et al., 2005). However, in the context of inflammatory bowel disease (IBD), existing research on fear of recurrence has predominantly focused on the diagnosis and treatment of mental illnesses, with a notable absence of systematic positive psychological interventions. Therefore, the scope of research should be expanded to thoroughly address psychological issues and facilitate the transition from traditional psychology to the field of psychological medicine.

The PERMA model, which encompasses positive affect (P), engagement (E), relationships (R), meaning (M), and achievement (A)—collectively referred to as “happiness PERMA”—has been widely recognized as a significant advancement in the field of positive psychology. Specifically, self-management training based on this model has typically been found to be more acceptable to patients than conventional, symptom-centric approaches to treatment. In addition, the PERMA model provides patients with ongoing psychological interventions that identify latent positive emotions, assist patients in confronting their illness and facilitate the correction of erroneous perceptions, thereby enhancing their quality of life and alleviating negative emotions. Currently, positive psychological interventions grounded in the PERMA model have demonstrated significant clinical efficacy in mitigating the fear of recurrence among patients with breast cancer (Chen et al., 2022). At the same time, these interventions have been shown to improve the happiness of autistic adults (Grosvenor et al., 2023). However, there is a paucity of research examining the applicability of this intervention model in addressing the fear of recurrence among patients with inflammatory bowel disease (IBD). Therefore, this study aims to evaluate the impact of positive psychological interventions based on the PERMA model on the fear of relapse in patients with IBD.

## 2 Materials and methods

### 2.1 Study design

The single-blind, randomized, controlled trial was conducted over a three-month period. The research protocol received approval from the Research Ethics Committee of the Affiliated Hospital of Jiangnan University (approval number: LS2024231) and was registered with the Chinese Clinical Trial Registry (ChiCTR2400085278). Reporting of the present trial follows CONSORT guidelines for randomized pilot and feasibility trials (Eldridge et al., 2016). In addition, written informed consent was obtained from all participants prior to their inclusion in the study. Randomization and grouping were performed by a research assistant who was blinded to the study design and protocol. This assistant utilized a computer-generated random number table to assign participants to either the intervention group (IG) or the control group (CG). The assignments were placed in sealed envelopes which were opened only after the baseline measurements were collected. However, due to the nature of the intervention, the study was blinded solely to the data collector.

### 2.2 Settings and participants

**Figure 1:**
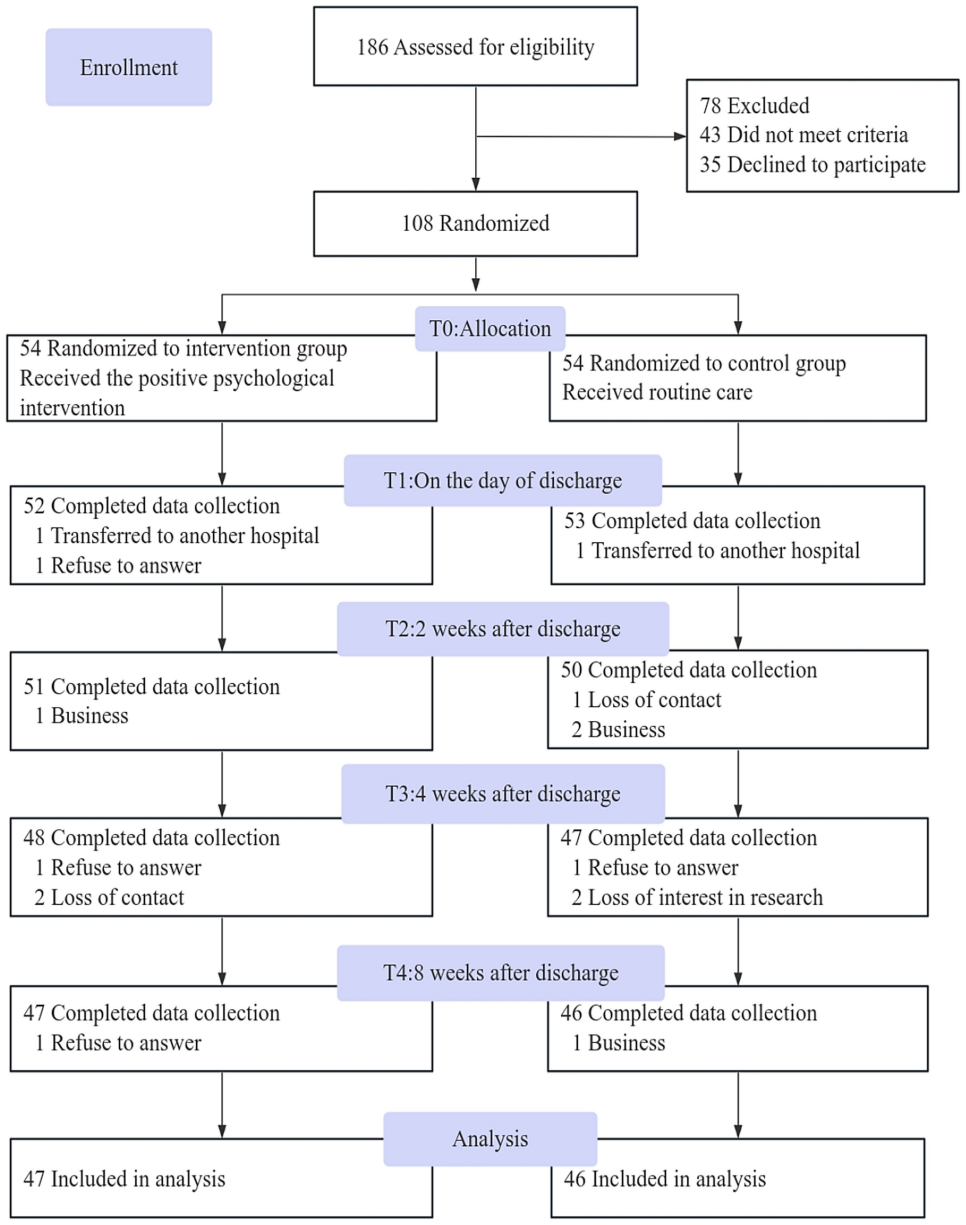
Flow chart of participants’ enrollment.

The study population was composed of 93 patients experiencing fear of recurrence, who were recruited from a tertiary hospital in Wuxi City (Figure 1). The inclusion criteria were as follows: (1) diagnosis meeting the criteria for UC or CD as proposed by the Chinese consensus on the diagnosis and treatment of inflammatory bowel disease (IBD) (Hu, 2012), and who require regular treatment with biological agents (including patients in remission and those in active phase). For UC, disease activity is assessed using the Wallemarius Simplified Clinical Colitis Activity Index (SCCAI) (Walmsley et al., 1998), while for CD, the Simplified Crohn’s Disease Activity Index (SCDAI) (Harvey and Bradshaw, 1980) is employed. The assessment criteria for these two activity indices are identical: when a patient’s score is ≤ 4 points, this indicates a remission period for the disease; (2) primary school education level or higher; (3) a score of ≥ 34 points on the Fear of Progression Questionnaire-Short Form (FoP-Q-SF), indicating a clinically significant level of fear; (4) proficiency in operating smartphones by the patients or their caregivers; and (5) voluntary participation in the study with signed informed consent. Similarly, the exclusion criteria included: (1) severe heart, lung, liver, or kidney diseases, or malignant tumors; (2) legal blindness or severe visual impairment; and (3) mental disorders or cognitive impairments.

### 2.3 Control group

The control group was provided with standard nursing care, which consisted of the following components: (1) educational knowledge delivered to patients with inflammatory bowel disease (IBD); (2) pharmacological treatment administered to IBD patients; (3) nutritional guidance offered to IBD patients; (4) psychological support provided to IBD patients; and (5) daily living nursing care carried out for IBD patients.

### 2.4 Intervention group

The study design included a control group that received a standard-of-care intervention, which was implemented in the Department of Gastroenterology. The intervention team was managed by a ten-member multidisciplinary research team consisting of a graduate supervisor, two professors of gastroenterology, a mental health specialist, two gastroenterology residents, a rehabilitation specialist, a head nurse, and two nurses from the gastroenterology department. The graduate supervisor was responsible for overseeing the overall learning design. Similarly, mental health specialists provided unified guidance and training for the intervention, coordinated efforts with head nurses, and managed patient health education. Graduate students, in collaboration with nursing staff, implemented nursing interventions, conducted interviews, and performed data collection and analysis. Finally, all members of the intervention team completed specialized training in positive psychological care to ensure the quality and consistency of the intervention.

Development of the Intervention Program: The intervention program was formulated based on a review of relevant literature from the past 5 years regarding the psychology of fear of recurrence and psychological interventions using the PERMA model for patients with inflammatory bowel disease (IBD). Specifically, the review aimed to understand the levels of fear, hope and happiness in people with IBD and to assess the effects of these interventions. Subsequently, an initial positive psychological intervention program was developed using the PERMA model, which was refined through two rounds of expert consultations via email or face-to-face meetings, with adjustments based on expert feedback.

Implementation of the intervention: The intervention was structured around the five themes of the PERMA model and was delivered through twelve staged sessions.

1. First Session (Within 3 Days of Admission): The theme of the session was “Injecting Positive Emotions.” A one-on-one session lasting 30 to 45 min was held at the bedside, using PowerPoint as a visual aid. In addition, WeChat groups were set up to include the friends of patients, fostering a relationship of mutual trust between patients and their social circle.
2. Second Session (Within 4–7 Days of Admission): The theme was “Immersion in the Flow State.” The 30- to 45-min session was designed to facilitate patients’ experience of a “flow state,” diverting their attention from stress and physical discomfort through immersive relaxation training.
3. Third Session (Days 8–10 of Hospitalization): The theme of this session was “Creating and Preserving Positive Relationships.” The agenda included a group sharing session lasting 30 to 45 min, which involved patients, family members, and professionals. In this group setting, participants engaged in self-disclosure, shared their feelings, and learned communication skills through scenario simulations and role reversals.
4. Fourth Session (Day of Discharge): The theme was “Actively Understanding the Meaning of Life.” The 30- to 45-min session focused on affirming patients’ self-worth, encouraging active participation in life, and collaboratively planning post-discharge rehabilitation exercises.
5. Fifth Session (Within a Week of Discharge): The theme for this session was “Understanding the Meaning of Life.” Delivered through a 10- to 15-min web video, this session was designed to sustain patient engagement in their rehabilitation program while offering timely feedback for necessary adjustments.
6. Sixth Session (Within 2 Weeks of Discharge): The theme continued to be “Understanding the Meaning of Life.” This 10- to 15-min online video session was designed to reinforce the significance of adhering to a structured recovery program while emphasizing the positive aspects of patient progress.
7. Seventh Session (Within 3 Weeks of Discharge): The theme was again “Understanding the Meaning of Life.” The 10- to 15-min online video session was designed to ensure that patients actively follow their recovery plan. Patients were encouraged to share videos of their daily lives in a WeChat group to foster a sense of community and positive reinforcement.
8. Eighth Session (Within 4 Weeks of Discharge): The theme was “Achieving a Sense of Accomplishment.” This 15- to 20-min online video session focused on helping patients set viable life goals and actively pursue them. Patients were encouraged to continue their daily relaxation training and share their immersive experiences in a WeChat group to maintain motivation and a sense of achievement.
9. Ninth Session (Within 5 Weeks of Discharge): The theme was “Consolidating Positive Changes.” This 15- to 20-min online video session reviewed progress over the past 5 weeks, highlighting physical improvements, emotional stability, and positive lifestyle changes. Patients engaged in self-motivation training by setting personal growth goals and participated in family activities to enhance emotional bonds and social support.
10. Tenth Session (Within 6 Weeks of Discharge): The theme for this session was “Managing Emotions and Enhancing Social Interaction.” This 15- to 20-min online video session included training in mood management through techniques such as mood journaling and progressive relaxation. Patients were encouraged to participate in online exchanges with other patients and develop family support plans, thereby strengthening their social support networks and effectively coping with mood swings.
11. Eleventh Session (Within 7 Weeks of Discharge): The theme for this session was “Establishing a Healthy Lifestyle.” This 15- to 20-min online video session offered education on healthy living, covering essential topics such as diet, exercise, and sleep. Patients learned self-management skills, including how to monitor their health status and adjust their rehabilitation program accordingly. In addition, they were encouraged to engage in community activities to enhance social integration and improve their overall quality of life.
12. Twelfth Session (Within 8 Weeks of Discharge): The theme for this session was “Looking Forward and Preparing for Reintegration.” This 30- to 45-min online video session involved a comprehensive review of the eight-week recovery journey, highlighting the achievements and lessons learned. Patients set future goals, planned for their reintegration, and shared their experiences in community or online support groups to strengthen social connections and prepare for the next phase of their lives.

Throughout the intervention period, medical staff provided supervision and guidance via WeChat, telephone or monthly face-to-face consultations. See Table 1 for further details.

**Table 1:** Positive psychological intervention program of PERMA model.

Time	Topic	Intervention goals	Content of the intervention
Stage 1: (Admission to hospital 1–3 days)	Inject positive emotions	(1) Build a relationship of trust. (2) Assessing the psychological problems of fear of relapse. (3) Explain the positive effect and value of communication output. (4) Transfer positive energy.	One-on-one intervention in the form of bedside IPD PowerPoint (30–45 min): (1) Introduce yourself, answer their questions about hospitalization, and help them solve the problems they are facing to enhance mutual trust. (2) Assess the patients, clarify the patients’ views and cognition of their own disease, and the problems they face, and then hold relevant knowledge lectures to answer these problems. (3) To assess the physical or mental health problems of IBD patients with fear of recurrence. To illustrate the power of positive emotions, guide and sort out the daily emotion management, put forward the solution to establish positive emotions, lead to PERMA theory, introduce the concept, methods and benefits. Choose to write down phrases (including but not limited to) about courage. a. Each treatment is a step towards better health, and I will do it. b. Illness is only a temporary challenge. I will overcome it. c. I use persistence to pave the way for health, never give up easily. d. Be strong: Be strong as a rock, and you will regain your health.
Stage 2: (Admission to hospital 4–7 days)	Immersion in the flow state	(1) Using “blessing flow” to divert attention and relieve stress and physical discomfort. (2) Patients were required to focus on completing relaxation technique training at least twice a day, and punch in WeChat group to share immersive experience.	The intervention was conducted one-on-one at the bedside (30-45 min): (1) According to the patient’s personalized hobbies, activities such as singing, playing chess, reading, painting, handwork, and listening to music were carried out to lead to the definition and positive effect of “flow” experience; The needs and physical conditions of patients were assessed: interests and needs, so as to choose suitable activities and methods. After the activities, patients were organized to share their experiences and feelings. (2) Training of guided relaxation techniques, deep breathing exercises: Teaching patients the method of deep breathing, let them practice during or after the activity, guide the patient to feel the changes in breathing, relax the various parts of the body. (3) Put in the experience of blessing flow, and train under the guidance of the psychologist: play light music as the background of Ballade For Adeline, and use progressive muscle relaxation. Sit or lie down in a quiet, comfortable place and close your eyes. a. Tense muscles: Start at the foot, gradually tighten the muscles and hold for 5–10 s to feel the muscle tension. b. Relax your muscles: Then suddenly relax and feel the relaxation of your muscles. Leave for 15–20 s. c. Step by step: Perform the same tension-relaxation exercises on the lower legs, thighs, buttocks, abdomen, chest, arms, neck, and face in sequence.
Stage 3: (Admission to hospital 8–10 days)	Creating and preserving positive relationships	(1) Understand the benefits of positive communication. (2) Learn communication skills through situational simulation and transposition.	Focus on face-to-face interactive explanations in the demonstration classroom, in the form of group sharing sessions (30–45 min): (1) To deeply understand the current interpersonal relationship of patients and the communication state between patients and their families, and explain the skills of interpersonal relationship establishment and the importance of communication. (2) The medical team went deeply into the patient and paid attention to the patient’s concerns; Let it show its heart. (3) Each group consisted of patients, family members and professionals, who shared their inner feelings in a group form. (4) Scenario simulation: training communication skills to deal with stressful situations: asking patients and their families to say to each other suggestions for improvement in communication, encouraging patients and their families to reveal their true feelings, express their understanding and views of disease recurrence, and talk to each other; Guide both sides to put themselves in other’s shoes and understand the efforts made by both sides to overcome the disease. For example, the health care leader said to the patient, “Now please share your understanding and feelings about the recurrence of the disease. You can talk about your worries, fears, and hopes for the future.” When the patient had finished speaking, the facilitator turned to the family: “Now please share your feelings after hearing these words and your thoughts on the recurrence. You can talk about your anxiety, helplessness, or you can talk about your support and encouragement to the patient.”
Stage 4: Day of discharge	Actively understanding the meaning of life	(1) Affirm their own value, actively participate in life, feel the current time; Words of encouragement to fellow IBD patients. (2) The post-discharge diet and nutrition plan was formulated.	Bedside face-to-face intervention (30–45 min): (1) Face the disease optimistically and face up to the current physical state. (2) Family members were invited to communicate with psychotherapists and nutritionists to teach positive understanding of the meaning of life. A discharge message booklet was distributed, and a paragraph was written to encourage IBD patients to share their happiness and experience the joy of helping others. Or record a video encouraging the patient to share the joy. For example: “Dear patient, you have taken the first step in your recovery. Please believe that every effort is an important step towards health. We are always with you and look forward to your regaining confidence and joy in life.” (3) Correct and reasonable living habits and work with a dietitian to develop an individualized adherence diet therapy plan.
Stage 5: 1 week after discharge	Understanding the meaning of Life	(1) Set realistic life goals and work hard to pursue them. (2) Timely feedback and adjustment of diet and nutrition treatment plan. (3) Patients were required to focus on completing relaxation technique training at least twice a day, punch in WeChat group, and share immersive experience.	Online video interventions (15–20 min): (1) Encourage patients to continue to maintain good living habits. (2) Encouraging family members to give encouraging words at home, providing a good family atmosphere for patients and family members to exchange encouraging words. You are always the most important person in this family. We are all rooting for you and praying for you. You can take care of your illness and leave the rest to us. Our family will never be apart (3) Help patients to set feasible goals, and then strive to pursue these goals, in the process of achieving goals, can feel their own progress and achievement.
Stage 6: 2 weeks after discharge		(1) Orderly and active relaxation training plan according to the plan; And share it on WeChat. (2) Confidence and firmness to complete the set goal. (3) Timely feedback and adjustment plan.	Online video interventions (15–20 min): (1) Immersive self-training according to the relaxation plan. (2) Patients were encouraged to complete one favorite activity every day or every 2 days, including radio exercises, calligraphy, singing, food production, reading, etc., and to share and feel in WeChat groups.
Stage 7: 3 weeks after discharge		(1) Orderly and active exercise according to the relaxation training plan. (2) Confidence to complete the goal. (3) The sense of accomplishment spreads to others. (4) Timely feedback and adjustment.	Online video interventions (15–20 min): (1) Give the patient the affirmation and support of the current living state, and correct the bad living habits in the process. (2) Guide patients to continue to do what they are good at and experience the sense of achievement brought by the use of advantages. (3) Recording the life experience; What you consider a small accomplishment. (4) Share your small achievements with your patients.
Stage 8: 4 weeks after discharge		(1) Consolidate the achievements and maintain a positive attitude towards life. (2) Enhance the sense of social participation and expand social support network; The sense of accomplishment is contagious. (3) Summing up experience to improve self-management ability; Timely feedback and adjustment plan.	Online video interventions (15–20 min): (1) Review of the training process: patients were invited to share the training experience of the past four weeks, including successes and challenges, and guided to summarize the lessons and enhance their self-efficacy. (2) Expanding social support: patients were encouraged to participate in community activities or online support groups to share their own stories, communicate with other patients, and enhance their sense of social integration. (3) Setting long-term goals: guiding patients to formulate training plans and life goals for the next three months, emphasizing the feasibility and sustainability of goals, and helping patients maintain a positive attitude towards life. (4) Family support and feedback: family members were invited to participate in online meetings to share the patient’s relaxation training at home, provide feedback and suggestions, and jointly adjust the plan to ensure the smooth implementation of the plan. (5) Recording life clips: patients were encouraged to record videos of themselves participating in community activities or family interactions and share them in WeChat groups or support groups to enhance their sense of accomplishment and belonging.
Stage 9: 5 weeks after discharge		(1) Consolidate rehabilitation achievements and continuously improve physical function and quality of life. (2) Cultivate the ability of self-reflection and self-motivation to promote personal growth. (3) Strengthen family and social support to enhance happiness and sense of belonging.	Online video interventions (15–20 min): (1) Review and summary: Review the rehabilitation process of the past 4 weeks, share the changes in physical function, emotional state, and life attitude, summarize experience, and find growth and progress. (2) Self-motivation training: make a personal growth plan, such as learning painting, calligraphy, handicrafts, reading helpful books, participating in online courses, etc. Set small goals and gradually achieve them, and experience the sense of accomplishment of self-improvement. (3) Family interactive activities: Design family interactive games or activities, such as family sports games, handmade, etc., to enhance the interaction and emotional connection between family members and feel the support and care of the family. (4) Social support expansion: participate in community volunteer service activities, such as helping the elderly in the community, participating in environmental protection activities, etc., and enhance the sense of social value and happiness by helping others.
Stage 10: 6 weeks after discharge		(1) Maintain a positive attitude and cope with emotional fluctuations in the process of rehabilitation. (2) Improve the ability of emotion management, learn to deal with stress and frustration. (3) Strengthen the interaction with the society and enhance the sense of social participation.	Online video intervention (15–20 min): (1) Emotion management training: identify and express emotions and learn emotion regulation skills, such as progressive relaxation training and mindfulness meditation, through emotional diary and emotional expression. (2) Positive attitude cultivation: Share stories or cases of positive psychology, view difficulties and challenges in the process of rehabilitation from a positive perspective, and cultivate an optimistic attitude. (3) Social interaction activities: participate in online patient communication meetings, invite patients with better recovery to share their experiences, encourage and support each other, and enhance the social support network. (4) Family support plan: Family members were invited to participate in online meetings to discuss how to better support the patient’s emotional management and formulate family support plans, such as regular family gatherings and joint activities.
Stage 11: 7 weeks after discharge		(1) To establish a healthy lifestyle and consolidate the rehabilitation results. (2) Improving self-management ability to ensure the sustainability of the rehabilitation program. (3) To promote the integration with society and improve the quality of life.	Online video interventions (30–45 min): (1) Healthy lifestyle education: learn the knowledge of healthy diet, regular work and rest, moderate exercise and other aspects, and formulate personalized healthy life plan, such as reasonable diet arrangement and exercise plan. (2) Improvement of self-management ability: learn to self-monitor physical condition, such as regular weight measurement, blood routine, etc., record rehabilitation process, and timely adjust rehabilitation plan. (3) Social integration activities: participate in community activities or interest groups, such as Tai chi, yoga, calligraphy association, etc., to improve the quality of life and enhance the sense of social integration by participating in social activities. (4) Family support and feedback: family members were invited to participate in online meetings to share the patient’s rehabilitation at home, provide feedback and suggestions, and jointly adjust the rehabilitation plan to ensure the smooth implementation of the rehabilitation plan.
Stage 12: 8 weeks after discharge		(1) Review the rehabilitation process, summarize the experience and lessons, and enhance self-efficacy. (2) Making future rehabilitation plans and life goals, and maintaining a positive attitude towards life. (3) Strengthen family and social support to prepare for returning to society.	Online video interventions (30–45 min): (1) Review of the rehabilitation process: sharing the rehabilitation experience of the past seven weeks, including successes and challenges, summarizing the lessons learned, and enhancing self-efficacy. (2) Future planning: make future rehabilitation plans and life goals, emphasize the feasibility and sustainability of the goals, and maintain a positive attitude towards life. (3) Family support and feedback: family members were invited to participate in online meetings to share the patient’s rehabilitation at home, provide feedback and suggestions, and jointly adjust the rehabilitation plan to ensure the smooth implementation of the rehabilitation plan. (4) Social support expansion: participating in community activities or online support groups, sharing recovery stories, and communicating with other recovery patients to enhance their sense of social integration. (5) Summary and outlook: sharing the outlook and expectation for the future, welcoming the new life with a positive attitude, and sending blessings and encouragement to the patients at the same time, ending the whole intervention plan.

### 2.5 Data collection and outcomes measures

This study utilized live surveys with researchers trained in standardized instructional language to facilitate participant engagement. Specifically, the study objectives and key points were effectively communicated to the researchers. Additionally, patients with inflammatory bowel disease (IBD) completed questionnaires independently and anonymously during the site visit, which were subsequently subjected to quality control by a designated officer. During the online follow-up intervention, follow-up nurses collected weekly data on the frequency and duration of patients’ online log-ins.

The Fear of Progression Questionnaire-Short Form (FoP-Q-SF) was developed by Mehnert et al. (2006), based on the original FoP-Q, and was subsequently adapted for the Chinese population by Wu et al. (2015). This simplified scale encompasses two dimensions: physical health and social family function, each consisting of six items, resulting in a total of twelve items. Using a 5-point Likert scoring method, each item is rated on a scale of 1 to 5, where 1 indicates “strongly disagree” and 5 indicates “strongly agree.” Total scores range from 12 to 60, with higher scores reflecting greater levels of fear experienced by patients. A total score of 34 or higher suggests that patients exhibit a fear of recurrence following inflammatory bowel disease (IBD). The Cronbach’s *α* coefficient for this scale exceeds 0.88, indicating good internal consistency.

The Subjective Well-Being Scale, developed by Campbell (1976) and later translated into Chinese by Fan (1999), is utilized to assess a patient’s subjective well-being. The scale consists of two sub-scales: the General Affective Index Scale, which includes eight items, and the General Life Satisfaction Questionnaire, which includes one item. The scoring range for this scale spans from 2.1 to 14.7, with lower scores (2.1–6) indicating low well-being, moderate scores (6.1–10) reflecting moderate well-being, and higher scores (10.1–14.7) signifying high well-being. A higher total score on the scale correlates with greater subjective well-being, and the scale exhibits strong reliability, evidenced by a Cronbach’s *α* coefficient of 0.90.

Resilience Scale for Inflammatory Bowel Disease (RS-IBD) was developed by Luo (2018). This questionnaire consists of 25 items categorized into six dimensions: self-management on disease (four items), active response to difficulty (six items), positive cognition (five items), emotional regulation (four items), family support (three items) and other patients’ support (three items). A 5-point Likert scale was used for scoring, where 0 means “never” and 4 means “always.” The total score ranges from 0 to 100, with higher scores indicating improved mental resilience. The Cronbach’s *α* coefficient for the RS-IBD scale is reported to be 0.94.

The Inflammatory Bowel Disease Quality of Life Questionnaire (IBDQ), developed by Guyatt et al. (1989), and translated into Chinese by Zhou and Lu (2004). Comprises 32 items divided into four dimensions: bowel symptoms (B) (10 items), systemic symptoms (S) (5 items), emotional function (E) (12 items), and social function (SF) (5 items). A 7-point Likert scale was utilized, where 1 denotes “very serious” and 7 indicates “normal.” Total scores range from 32 to 224, with higher scores indicating a better quality of life. The Chinese version of the IBDQ demonstrates a Cronbach’s *α* coefficient of 0.95.

### 2.6 Statistical analysis

IBM SPSS Statistics 27.0 (IBM SPSS, New York, United States) was utilized for data analysis in this study. Measurement data that conform to a normal distribution are represented as means and standard deviations, while the comparison between the two groups is analyzed using independent sample *t*-tests. For data that did not conform to normal distribution, results were presented as median (P_25_, P_75_), and the Mann–Whitney U test was employed for group comparisons. The categorical data are reported as counts or percentages, and group comparisons are analyzed using chi-square tests. Furthermore, this study employed the Generalized Estimating Equation (GEE) model to compare the overall outcome evaluation indicators between the two groups, examining differences and trends over time. The main effects of group and time, and their interplay, were investigated. A two-sided test was applied, with a significance level set at *p* < 0.05.

### 2.7 Sample size

The sample size estimation formula for comparing the means of two independent samples is given by: n1 = n2 = 2[(μ_α_ + μ*_β_*)*σ*/*δ*]^2^. Here, the significance level is set at two-sided α = 0.05, and the power of the test is 1 − β = 0.90, with β = 0.10. In this formula, σ represents the combined standard deviation, while δ denotes the difference between the two sample means. The values for μ_α_ and μ_β_ are provided as μ_α_ = 1.96 and μ_β_ = 1.282, respectively. Based on findings from similar studies, we have σ = 6.24 and δ = 4.96 (Frangou et al., 2021). Consequently, the calculated sample size is n1 = n2 = 33. Considering a projected loss rate of 30%, the final sample size required was 94 cases, with 47 allocated to the intervention group and 47 to the control group.

## 3 Results

### 3.1 Participant flow

A total of 186 patients were recruited for this study, of whom 108 met the inclusion criteria and were randomly assigned to either the intervention group (*n* = 54) or the control group (*n* = 54). Among them, 93 patients [intervention group (*n* = 47/54, 87%), control group (*n* = 46/54, 85%)] completed the 8-week follow-up, resulting in a loss to follow-up rate of 13.89% (15 patients). Specifically, the primary reasons for loss to follow-up included: 1. Patients were transferred to different hospitals for treatment due to their condition; 2. Refusal to participate due to work commitments and other reasons; 3. Loss of contact; and 4. Loss of interest in the study. Figure 1 shows the participant flow diagram.

### 3.2 Participant demographics and clinical characteristics

The mean age of the participants was 44.03 ± 14.99 years, with a mean cumulative number of recurrences of 5.67 ± 1.05. The majority of participants were male (62.37%) and married (73.12%). Approximately 46.24% of participants reported a per capita monthly household income of 3,000–5,000 RMB (US $418.04–696.7), and more than half indicated experiencing financial stress (59.14%). Participants were relatively well-educated, with 53.76 percent having a college education or higher and 66.67 percent employed by a business. Most participants (87.10%) resided in urban areas. All participants were covered by Medicare. More than half were diagnosed with ulcerative colitis upon admission (52.69%). Simultaneously, 55.91% of the participants experienced one or more related complications, including malnutrition, mild anal fistula and oral ulcers. The majority of diagnoses occurred more than 1 year prior (83.87%), and 82.80% had been hospitalized for inflammatory bowel disease more than five times. Additionally, the average BMI of participants was 18.04 ± 0.76, with most not smoking or drinking (68.82 and 69.98%, respectively), and a majority did not have hypertension, stroke, or other related chronic diseases (69.89 and 86.02%). There were no significant differences in age, gender, education level, occupational status, outpatient diagnosis, cumulative recurrence times, and disease status between the two groups (*p >* 0.05). Table 2 presents demographic information and clinical characteristics of the participants.

**Table 2:** Demographic and clinical traits of the participants.

Variables	Total (*n* = 93)	Intervention group (*n* = 47)	Control group (*n* = 46)	*t*/*χ*^2^*-*value	*p*-value
Age (Mean ± SD)	44.03 ± 14.99	43.04 ± 14.92	45.04 ± 15.17	−0.641^a^	0.523
Gender (*n*, %)				0.087^b^	0.832
Male	58 (62.37)	30 (63.83)	28 (60.87)		
Female	35 (37.63)	17 (36.17)	18 (39.13)		
Clinic diagnosis (*n*, %)				0.863^b^	0.353
UC	49 (52.69)	27 (57.45)	22 (47.83)		
CD	44 (47.31)	20 (42.55)	24 (52.17)		
Marital status (*n*, %)				0.125^b^	0.939
Married	68 (73.12)	35 (74.46)	33 (71.74)		
Unmarried	13 (13.98)	6 (12.77)	7 (15.22)		
Else*	12 (12.90)	6 (12.77)	6 (13.04)		
Status of occupation (*n*, %)				1.589^b^	0.452
Enterprises and institutions	62 (66.67)	31 (65.95)	31 (67.39)		
Worker/farmer	15 (16.13)	6 (12.77)	9 (19.57)		
Students and others	16 (17.20)	10 (21.28)	6 (13.04)		
Place of family residence (*n*, %)				0.002^b^	0.968
A city or town	81 (87.10)	41 (87.23)	40 (86.96)		
Rural area	12 (12.90)	6 (12.77)	6 (13.04)		
Degree of education (*n*, %)				1.245^b^	0.537
Primary school and below	16 (17.20)	7 (14.89)	9 (19.57)		
Junior/technical secondary/high school	27 (29.03)	16 (34.04)	11 (23.91)		
College or above	50 (53.76)	24 (51.06)	26 (56.52)		
Monthly household income per capita (Yuan) (*n*, %)				2.413^b^	0.491
<3,000	21 (22.58)	13 (27.66)	8 (17.39)		
3,000–5,000	43 (46.24)	22 (46.81)	21 (45.65)		
5,001–8,000	15 (16.13)	7 (14.89)	8 (17.39)		
>8,000	14 (15.05)	5 (10.64)	9 (19.57)		
Medical payment methods (*n*, %)				0.686^b^	0.877
Rural cooperative medical service	15 (16.13)	7 (14.89)	8 (17.39)		
The medical insurance for urban residents	12 (12.90)	5 (10.64)	7 (15.22)		
Employee’s medical insurance	54 (58.06)	29 (61.70)	25 (54.35)		
Others (at own or public expense)	12 (12.90)	6 (12.77)	6 (13.04)		
Duration of diagnosis (years) (*n*, %)				2.604^b^	0.302
<1	15 (16.13)	7 (14.89)	8 (17.39)		
1–3	40 (43.01)	24 (51.16)	16 (34.78)		
>3	38 (40.86)	16 (34.04)	22 (47.83)		
BMI (kg/m^2^, Mean ± SD)	18.04 ± 0.76	17.96 ± 0.69	18.13 ± 0.83	−1.054^a^	0.295
Cumulative number of recurrences (Mean ± SD)	5.67 ± 1.05	5.72 ± 1.08	5.61 ± 1.02	0.527^a^	0.600
Number of hospitalizations due to IBD (*n*, %)				0.002^b^	0.962
<5	16 (17.20)	8 (17.02)	8 (17.39)		
≥5	77 (82.80)	39 (82.98)	38 (82.61)		
Current disease status (*n*, %)				2.877^b^	0.090
Remission	79 (84.95)	37 (78.72)	42 (91.30)		
Active stage	14 (15.05)	10 (21.28)	4 (8.70)		
IBD-related complications (*n*, %)**				0.014^b^	0.907
No	41 (44.09)	21 (44.68)	20 (43.48)		
Yes	52 (55.91)	26 (55.32)	26 (56.52)		
Smoking (*n*, %)				1.414^b^	0.234
No	64 (68.82)	35 (74.47)	29 (63.04)		
Yes	29 (31.18)	12 (25.53)	17 (36.96)		
Alcohol drinking (*n*, %)				0.271^b^	0.603
No	65 (69.98)	34 (72.34)	31 (67.39)		
Yes	28 (30.11)	13 (27.66)	15 (32.61)		
Hypertensive disease (*n*, %)				0.005^b^	0.946
No	65 (69.89)	33 (70.21)	32 (69.57)		
Yes	28 (30.11)	14 (29.79)	14 (30.43)		
Coronary heart disease (*n*, %)				0.116^b^	0.733
No	80 (86.02)	41 (87.23)	39 (84.78)		
Yes	13 (13.98)	6 (12.77)	7 (15.22)		
Stroke (*n*, %)				0.116^b^	0.733
No	80 (86.02)	41 (87.23)	39 (84.78)		
Yes	13 (13.98)	6 (12.77)	7 (15.22)		
Self-assessment of economic stress (*n*, %)				4.985^b^	0.083
No Stress	12 (12.90)	5 (10.64)	7 (15.22)		
Some pressure	55 (59.14)	33 (70.21)	22 (47.83)		
Under great pressure	26 (27.96)	9 (19.15)	17 (36.96)		

* Other marital status included being divorced and widowed, ** IBD-related complications refer to the occurrence of one or more conditions, such as malnutrition, mild anal fistula and oral ulcers.

SD, standard deviation; UC, ulcerative colitis; CD, Crohn disease.

a Independent samples *t*-test.

b Chi-squared test.

### 3.3 Effect of intervention on fear of recurrence, subjective well-being

**Figure 2:**
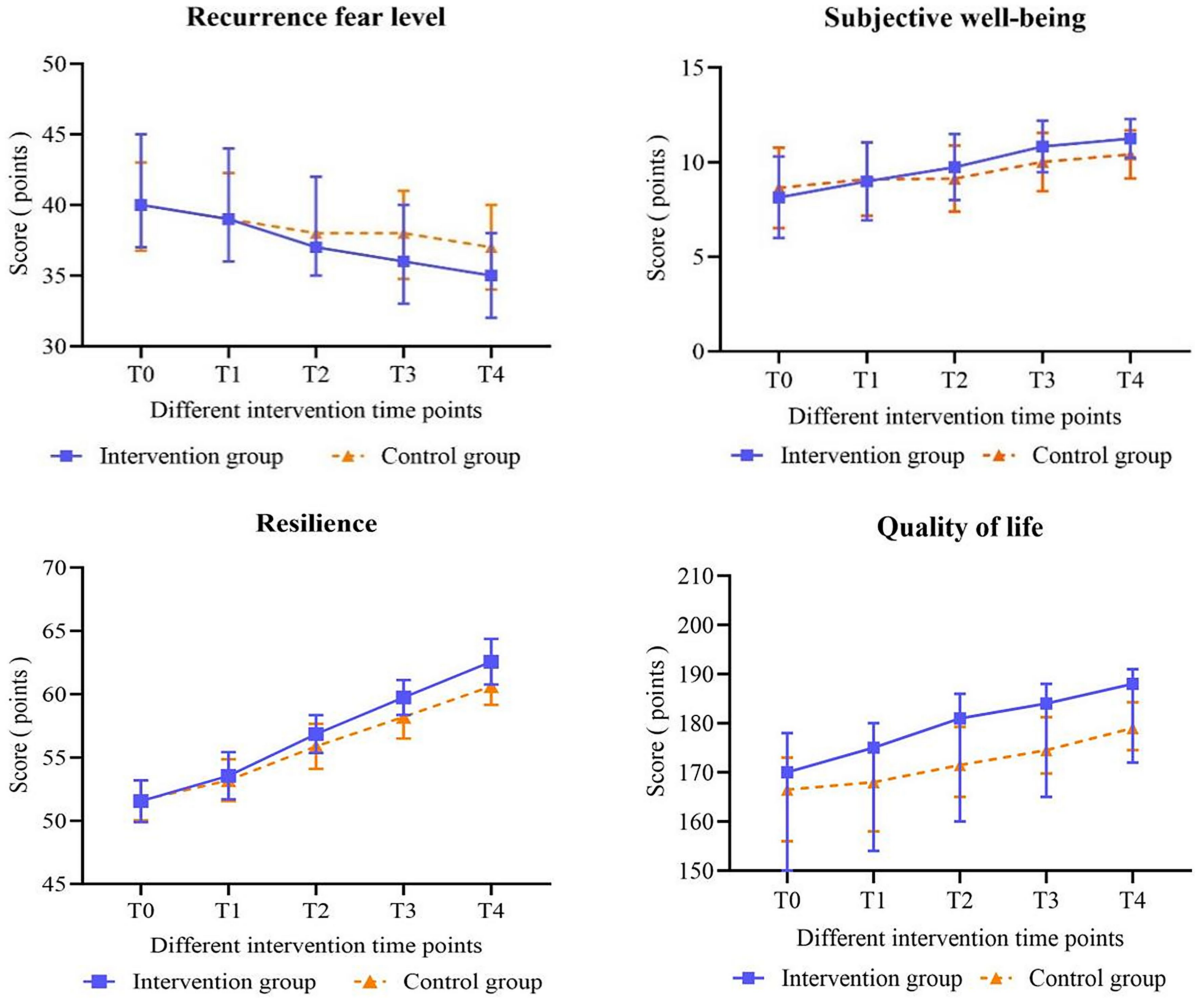
Changes in the scores of the fear of recurrence, subjective well-being, psychological resilience and quality of life of patients were analyzed.

Tables 3, 4 indicate that at T0 (baseline), there was no significant difference in the levels of fear of recurrence (*U* = −0.532, *p* = 0.595) and subjective well-being (*t* = −1.149, *p* = 0.254) between the patients in the intervention and control groups. In comparison to the control group, the intervention group exhibited lower levels of fear of recurrence at T3 (*U* = −1.978, *p* = 0.048) and T4 (*U* = −2.116, *p* = 0.034). Moreover, the CD patients in the intervention group had a decreased SCDAI scores at T4 (*U* = −2.160, *p* = 0.031) (see Table 5), alongside an increase in subjective well-being at T3 (*t* = 2.731, *p* = 0.008) and T4 (*t* = 3.490, *p* < 0.001), with these differences being statistically significant. However, no statistically significant differences were observed at the remaining intervention time points. Table 6 reveals that there were no significant differences in the levels of fear of recurrence and subjective well-being between the two groups (*χ^2^* = 0.273, *p* = 0.603; *χ^2^* = 0.925, *p* = 0.339), although both measures changed significantly over time (*χ^2^* = 240.207, *p* < 0.001; *χ^2^* = 71.870, *p* < 0.001). Additionally, there was a significant interaction between group and time (*χ^2^* = 27.016, *p* < 0.001; *χ^2^* = 15.274, *p* < 0.001). Figure 2 illustrates the changes in scale scores for fear of recurrence and subjective well-being in both groups across different intervention time points.

**Table 3:** Comparison of recurrence fear scores between the two groups.

Variable	Time	Intervention group M (P_25_, P_75_)	Control group M (P_25_, P_75_)	*U-*value	*P-*value
Fear of recurrence	T0	40.00 (37.00, 45.00)	40.00 (36.75, 43.00)	−0.532	0.595
	T1	39.00 (36.00, 44.00)	39.00 (36.00, 42.25)	−0.459	0.646
	T2	37.00 (35.00, 42.00)	38.00 (35.00, 42.00)	−0.551	0.581
	T3	36.00 (33.00, 40.00)	38.00 (34.75, 41.00)	−1.978	0.048
	T4	35.00 (32.00, 38.00)	37.00 (34.00, 40.00)	−2.116	0.034

M (P_25_, P_75_) median, quartiles.

**Table 4:** Comparison of well-being index scores between the two groups.

Variable	Time	Intervention group (Mean ± SD)	Control group (Mean ± SD)	*t-*value	*P-*value
Subjective well-being	T0	8.14 ± 2.15	8.64 ± 2.12	−1.149	0.254
	T1	8.99 ± 2.06	9.10 ± 1.93	−0.275	0.784
	T2	9.74 ± 1.75	9.13 ± 1.75	1.585	0.116
	T3	10.83 ± 1.36	10.01 ± 1.54	2.731	0.008
	T4	11.25 ± 1.02	10.41 ± 1.27	3.490	<0.001

**Table 5:** Comparison of disease activity scores between the two groups.

Variable	SCCAI Scores						SCDAI Scores					
	Number of cases	T0	T1	T2	T3	T4	Number of cases	T0	T1	T2	T3	T4
Intervention group	27	3.00 (2.00, 4.00)	2.00 (1.00, 4.00)	3.00 (1.00, 4.00)	2.00 (1.00, 5.00)	2.00 (1.00, 4.00)	20	4.00 (3.00, 4.75)	3.00 (2.00, 4.00)	2.00 (1.25, 5.50)	2.50 (2.00, 5.50)	2.00 (1.00, 3.75)
Control group	22	3.50 (3.00, 4.00)	3.00 (2.00, 5.00)	3.00 (2.00, 4.00)	3.00 (2.00, 5.25)	2.50 (2.00, 3.25)	24	3.00 (2.25, 4.00)	3.00 (2.25, 5.00)	2.5 (2.00, 5.00)	2.5 (2.00, 5.75)	2.5 (2.00, 5.00)
*U*-value		−0.200	−1.109	−0.031	−1.928	−0.249		−1.850	−0.624	−1.172	−0.139	−2.160
*P*-value		0.842	0.268	0.975	0.054	0.804		0.064	0.532	0.241	0.890	0.031

SCCAI, Simplified Clinical Colitis Activity Index; SCDAI, Simplified Crohn’s Disease Activity Index.

**Table 6:** The effects of generalized estimating equation model on fear of recurrence, subjective well-being, resilience, and quality of life were tested.

Variables	Effect	Wald *χ^2^*	*P-*value
Fear of recurrence	Group	0.273	0.603
	Time	240.207	<0.001
	Group*Time	27.016	<0.001
Subjective well-being	Group	0.925	0.339
	Time	71.870	<0.001
	Group*Time	15.274	<0.001
Resilience	Group	14.353	<0.001
	Time	186.089	<0.001
	Group*Time	6.080	<0.001
Self-management on disease	Group	3.522	0.064
	Time	177.782	<0.001
	Group*Time	3.753	0.007
Active response to difficulty	Group	2.155	0.146
	Time	40.414	<0.001
	Group*Time	2.499	0.048
Positive cognition	Group	4.738	0.032
	Time	144.884	<0.001
	Group*Time	1.281	0.284
Emotional regulation	Group	1.889	0.173
	Time	150.611	<0.001
	Group*Time	1.375	0.249
Family support	Group	2.327	0.131
	Time	181.040	<0.001
	Group*Time	0.368	0.831
Other patients’ support	Group	2.171	0.144
	Time	217.324	<0.001
	Group*Time	0.457	0.767
Quality of life	Group	0.698	0.403
	Time	84.677	<0.001
	Group*Time	4.035	0.005
Bowel symptoms	Group	1.062	0.306
	Time	12.198	<0.001
	Group*Time	2.344	0.062
Systemic symptoms	Group	0.992	0.319
	Time	44.425	<0.001
	Group*Time	1.748	0.147
Emotional function	Group	0.898	0.343
	Time	61.010	<0.001
	Group*Time	20.938	<0.001
Social function	Group	0.194	0.660
	Time	172.252	<0.001
	Group*Time	7.197	<0.001

### 3.4 Impact of the intervention on resilience

**Figure 3:**
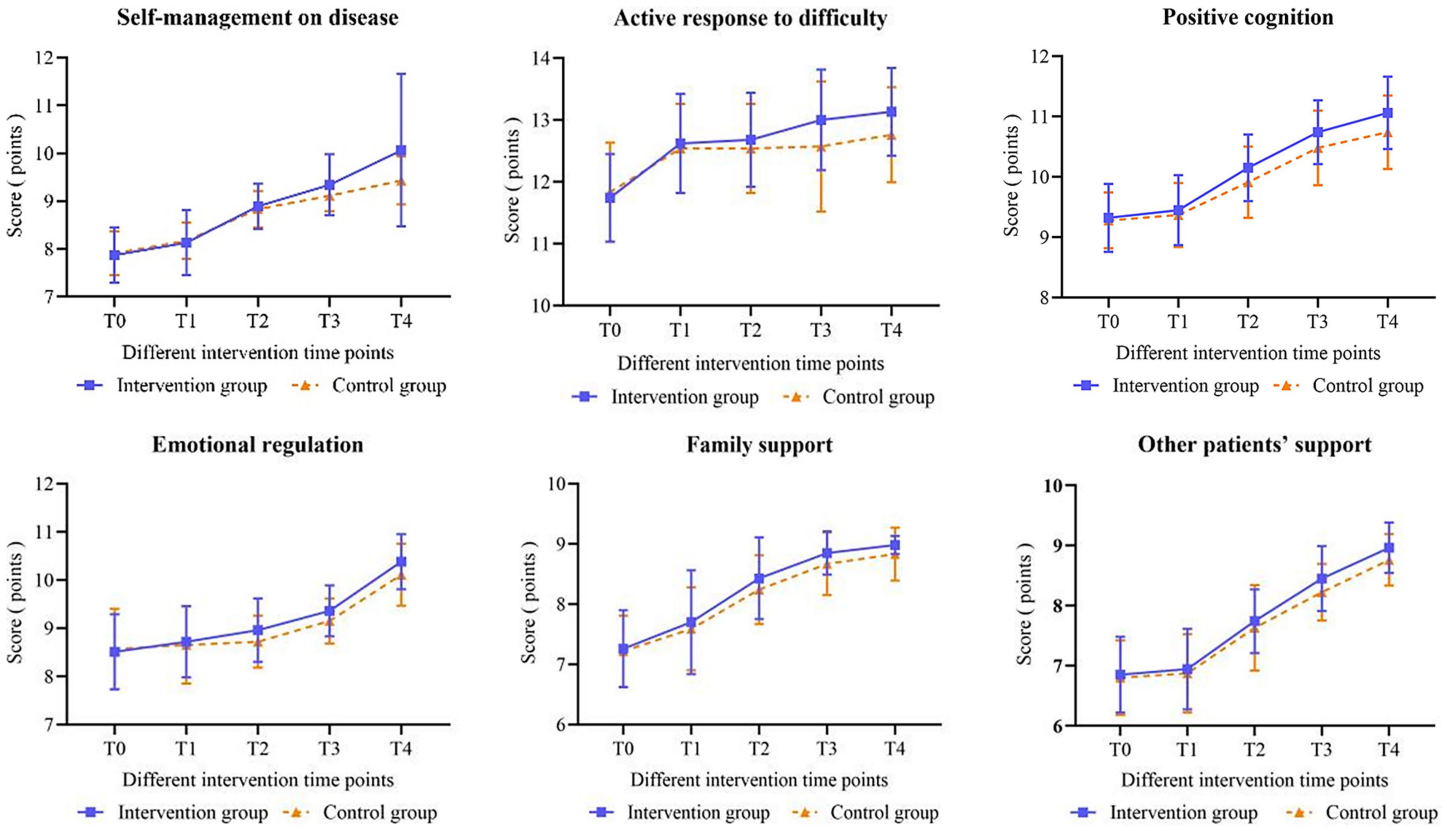
Changes in the mean scores of the six dimensions of resilience in patients.

Table 7 indicates that at T0 (baseline) and T1, there were no statistically significant differences between the total resilience scores and the scores across the six dimensions (self-management on disease, active response to difficulty, positive cognition, emotional regulation, family support and other patients’ support) when comparing patients in the intervention group to those in the control group (all *p* > 0.05). At T2, the scores on each dimension and the total score on the scale were found to be significantly higher in the intervention group than in the control group. Notably, the positive cognition dimension score (*t* = 1.994, *p* = 0.049) and the total score (*t* = 2.907, *p* = 0.005) demonstrated significant differences, while no significant differences were observed in the scores of other dimensions (all *p* > 0.05). At T3, the score for the family support dimension did not show a significant difference between the intervention and control groups (*t* = 1.917, *p* = 0.058); however, significant differences were noted in the scores of the additional five dimensions and the total score (all *p* < 0.05). At T4, statistically significant differences were observed in the total score of psychological resilience and in the scores of each dimension between the two groups (all *p* < 0.05). Table 6 illustrates significant intergroup effects in the positive cognition dimension scores and the total psychological resilience scores between the two groups (*χ^2^* = 4.738, *p* = 0.032; *χ^2^* = 14.353, *p* < 0.001). Notably, the total resilience score and the scores across six dimensions exhibited significant differences over time (all *p* < 0.001). The interaction between group and time had statistically significant effects on both the total resilience score and the score for active response to difficulty (*χ^2^* = 6.080, *p* < 0.001; *χ^2^* = 2.499, *p* = 0.048). Figures 2, 3 depict the changes in scores on the Psychological Resilience Scale and six dimensions at various intervention time points for both groups.

**Table 7:** Comparison of psychological resilience scores between the two groups.

Variables	Time	Intervention group (Mean ± SD)	Control group (Mean ± SD)	*t-*value	*P-*value
Total	T0	51.55 ± 1.65	51.61 ± 1.57	−0.166	0.869
	T1	53.55 ± 1.86	53.20 ± 1.65	0.978	0.331
	T2	56.85 ± 1.50	55.87 ± 1.78	2.907	0.005
	T3	59.74 ± 1.37	58.20 ± 1.71	4.824	<0.001
	T4	62.57 ± 1.80	60.63 ± 1.47	5.699	<0.001
Self-management on disease	T0	7.87 ± 0.58	7.91 ± 0.46	−0.375	0.708
	T1	8.13 ± 0.68	8.17 ± 0.38	−0.403	0.688
	T2	8.89 ± 0.48	8.83 ± 0.38	0.752	0.454
	T3	9.34 ± 0.64	9.11 ± 0.32	2.222	0.029
	T4	10.06 ± 1.59	9.43 ± 0.50	2.556	0.012
Active response to difficulty	T0	11.74 ± 0.71	11.83 ± 0.80	−0.522	0.603
	T1	12.62 ± 0.80	12.54 ± 0.72	0.467	0.642
	T2	12.68 ± 0.76	12.54 ± 0.72	0.897	0.372
	T3	13.00 ± 0.81	12.57 ± 1.05	2.246	0.027
	T4	13.13 ± 0.71	12.76 ± 0.77	2.395	0.019
Positive cognition	T0	9.32 ± 0.56	9.28 ± 0.46	0.346	0.730
	T1	9.45 ± 0.58	9.37 ± 0.53	0.667	0.506
	T2	10.15 ± 0.55	9.91 ± 0.59	1.994	0.049
	T3	10.74 ± 0.53	10.48 ± 0.62	2.222	0.029
	T4	11.06 ± 0.60	10.74 ± 0.61	2.573	0.012
Emotional regulation	T0	8.51 ± 0.78	8.57 ± 0.83	−0.327	0.745
	T1	8.72 ± 0.74	8.65 ± 0.80	0.447	0.656
	T2	8.96 ± 0.66	8.72 ± 0.54	1.915	0.059
	T3	9.36 ± 0.53	9.15 ± 0.47	2.019	0.046
	T4	10.38 ± 0.57	10.11 ± 0.64	2.178	0.032
Family support	T0	7.26 ± 0.64	7.22 ± 0.59	0.296	0.768
	T1	7.70 ± 0.86	7.59 ± 0.69	0.714	0.477
	T2	8.43 ± 0.68	8.24 ± 0.57	1.432	0.156
	T3	8.85 ± 0.36	8.67 ± 0.52	1.917	0.058
	T4	8.98 ± 0.15	8.83 ± 0.44	2.267	0.026
Other patients’ support	T0	6.85 ± 0.63	6.80 ± 0.62	0.362	0.718
	T1	6.94 ± 0.67	6.87 ± 0.65	0.484	0.629
	T2	7.74 ± 0.53	7.63 ± 0.71	0.880	0.381
	T3	8.45 ± 0.54	8.22 ± 0.47	2.179	0.032
	T4	8.96 ± 0.42	8.76 ± 0.43	2.241	0.027

### 3.5 Impact of the intervention on quality of life

**Figure 4:**
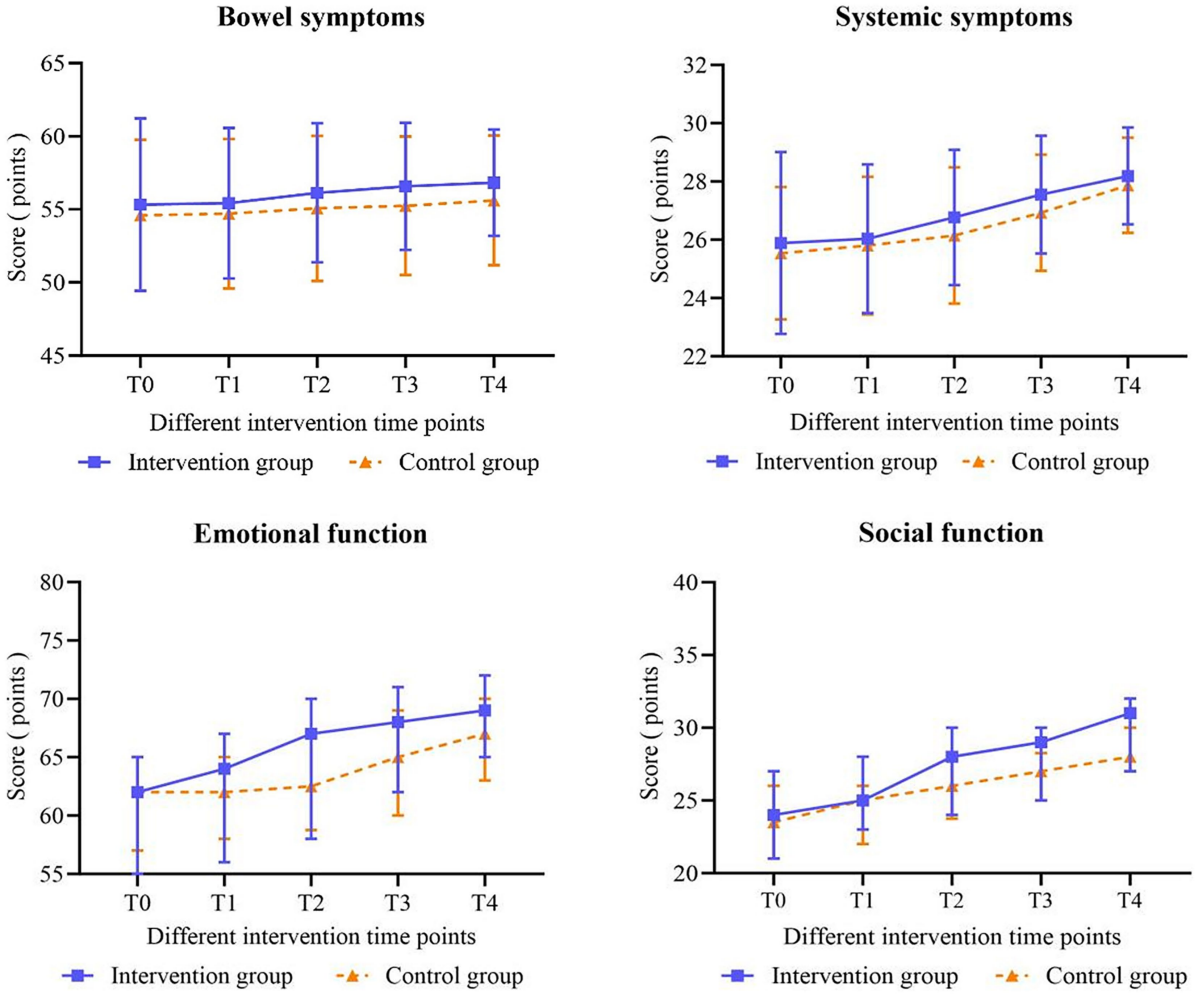
Changes in the scores of the four dimensions of the quality of life of patients were observed.

Table 8 illustrates that both bowel and systemic symptoms scores exhibited a gradual increase over time in both groups, with patients in the intervention group displaying slightly higher scores than those in the control group. However, none of the observed differences in changes between the groups reached statistical significance (all *p* > 0.05). Generalized estimating equations (GEE) presented in Table 6 further corroborate that the differences between the groups were not statistically significant, and there was no significant interaction between group and time regarding bowel symptoms scores (*χ^2^* = 1.062, *p* = 0.306) and systemic symptoms scores (*χ^2^* = 0.992, *p* = 0.319) for the two patient groups (respectively: *χ^2^* = 2.344, *p* = 0.062; *χ^2^* = 1.748, *p* = 0.147). At T2, T3, and T4, significant differences were observed between the two groups concerning scores for emotional competence, social ability, and overall quality of life (all *p* < 0.05). In the GEE model illustrated in Table 6, the scores for bowel symptoms, systemic symptoms, emotional function, social function, and total quality of life demonstrated significant differences over time (all *p* < 0.001). Additionally, a statistically significant interaction between group and time was identified for emotional function (*χ^2^* = 20.938, *p* < 0.001), social function (*χ^2^* = 7.197, *p* < 0.001), and the total quality of life score (*χ^2^* = 4.035, *p* = 0.005). Figures 2, 4 depict the changes in quality of life scale scores and the four dimensions of the scale for the two groups across different intervention time points, respectively.

**Table 8:** Comparison of quality of life scores between the two groups.

Variables	Time	Intervention group	Control group	*t*/*U-*value	*P-*value
Total M (P_25_, P_75_)	T0	170.00 (148.00, 178.00)	166.50 (156.00, 173.00)	−0.461^b^	0.645
	T1	175.00 (154.00, 180.00)	168.00 (158.00, 174.25)	−1.488^b^	0.137
	T2	181.00 (160.00, 186.00)	171.50 (165.00, 179.25)	−2.380^b^	0.017
	T3	184.00 (165.00, 188.00)	174.50 (169.75, 181.25)	−2.576_b_	0.010
	T4	188.00 (172.00, 191.00)	179.00 (174.50, 184.25)	−2.746^b^	0.006
Bowel symptoms (Mean ± SD)	T0	55.32 ± 5.89	54.59 ± 5.16	0.637^a^	0.526
	T1	55.43 ± 5.15	54.70 ± 5.11	0.686^a^	0.494
	T2	56.13 ± 4.76	55.07 ± 4.96	1.053	0.295
	T3	56.57 ± 4.34	55.24 ± 4.73	1.418^a^	0.160
	T4	56.83 ± 3.63	55.61 ± 4.43	1.455^a^	0.149
Systemic symptoms (Mean ± SD)	T0	25.89 ± 3.12	25.54 ± 2.27	0.618^a^	0.538
	T1	26.04 ± 2.55	25.80 ± 2.36	0.467^a^	0.642
	T2	26.77 ± 2.32	26.15 ± 2.34	1.270^a^	0.207
	T3	27.55 ± 2.02	26.93 ± 1.99	1.486^a^	0.141
	T4	28.19 ± 1.66	27.87 ± 1.63	0.943^a^	0.348
Emotional function M (P_25_, P_75_)	T0	62.00 (53.00, 65.00)	62.00 (57.00, 65.00)	−0.516^b^	0.606
	T1	64.00 (56.00, 67.00)	62.00 (58.00, 65.00)	−0.974^b^	0.330
	T2	67.00 (58.00, 70.00)	62.50 (58.75, 67.00)	−2.021^b^	0.043
	T3	68.00 (62.00, 71.00)	65.00 (60.00, 69.00)	−2.122^b^	0.034
	T4	69.00 (65.00, 72.00)	67.00 (63.00, 70.00)	−2.459^b^	0.014
Social function M (P_25_, P_75_)	T0	24.00 (21.00, 27.00)	23.50 (21.00, 26.00)	−0.058^b^	0.954
	T1	25.00 (23.00, 28.00)	25.00 (22.00, 26.00)	−0.939^b^	0.348
	T2	28.00 (24.00, 30.00)	26.00 (23.75, 28.00)	−2.152^b^	0.031
	T3	29.00 (25.00, 30.00)	27.00 (25.00, 28.25)	−2.268^b^	0.023
	T4	31.00 (27.00, 32.00)	28.00 (27.00, 30.00)	−2.494^b^	0.013

a Independent sample *t* test.

b Mann–Whitney U test.

## 4 Discussion

Patients with Inflammatory Bowel Disease (IBD) encounter numerous challenges and difficulties (Yu et al., 2021; Paulides et al., 2022). Specifically, they must navigate the unpredictable nature of flare-ups, the chronic and potentially debilitating course of the disease, and the risk of complications such as bowel perforation, constriction and malnutrition (Gecse and Vermeire, 2018). In addition, they regularly experience significant disruptions in their daily functioning, including limitations in physical activity, dietary restrictions and potential impacts on their social and occupational lives. Compared with individuals with other chronic conditions (Mattila et al., 2022; Gleave et al., 2025; Gravina et al., 2025), IBD patients are more prone to experiencing negative emotions such as helplessness, frustration, and self-doubt (Barberio et al., 2021). The constant focus on managing their illness leads to a perception that life is dominated by the disease, which diminishes their ability to live freely as they once did. Furthermore, chronic mental strain can lead to anxiety, depression and other psychological problems that undermine patients’ self-efficacy, reduce their sense of hope, increase feelings of vulnerability and erode their resilience.

This study represents the first randomized controlled trial to implement a positive psychology program based on the PERMA model in patients with IBD. The results showed that the intervention significantly alleviated patients’ fear of relapse, enhanced their psychological resilience, improved their subjective well-being, and ultimately improved their quality of life.

A recent meta-analysis in cancer and other chronic diseases has indicated that positive psychological interventions based on PERMA effectively reduce patients’ fear of relapse, thus supporting the findings of this study (Zhang et al., 2016). The PERMA framework is designed to promote positive emotions and optimism, which enhance patients’ overall perception of life and diminish disease-related fears. During the intervention, training that emphasized the development of close relationships and participation in social activities enabled IBD patients to establish meaningful goals, identify opportunities for personal growth, and strengthen their sense of responsibility to their families and society. Consequently, this approach fosters a more positive outlook on life and relieves the fear of relapse. Furthermore, in the advanced phase, personalized functional exercises and dynamic goal adjustment encourage IBD patients to focus on their strengths, explore their potential, and engage in self-healing practices. Ultimately, this, in turn, lessens the fear of relapse and enhances physical and social functioning. Research has suggested that activities such as progressive relaxation training, walking, and strength training can significantly improve the quality of life while reducing fatigue and stress among IBD patients (Mizrahi et al., 2012; Tiles-Sar et al., 2022; Sabir et al., 2024).

The findings of this study indicate that the experimental group exhibited significantly higher levels of psychological resilience post-intervention compared to the control group, a result consistent with previous research (Sirois and Hirsch, 2017; Sehgal et al., 2021). Therefore, this effect is most likely attributable to positive psychological interventions based on the PERMA model, which emphasizes the patient’s internal experiences and aims to enhance their positive cognitive appraisal of their illness. Specifically, the intervention team conducted a series of structured interviews aimed at correcting negative beliefs, addressing the psychological distress experienced by patients at various stages of their illness. In addition, the team facilitated opportunities for patients to engage in active communication with peers and family members, thereby promoting increased interpersonal relationships, self-esteem and confidence. This comprehensive approach is designed to progressively rectify patients’ misconceptions and negative perceptions related to their illness.

Patients were encouraged to express themselves through face-to-face interviews and written recordings. In addition, they also participated in stimulating activities such as singing aloud, playing chess, and engaging in other positive events. These activities were specifically designed to foster the development of a positive mindset (Yeh et al., 2016; Cano et al., 2020). Therefore, by enhancing beliefs and confidence, this approach aims to reduce symptoms of depression and improve overall mood, ultimately enhancing psychological resilience in patients with inflammatory bowel disease (IBD).

In recent years, the comprehensive application of positive psychology research has underscored the significance of well-being as a critical metric for assessing the psychological state of individuals with Inflammatory Bowel Disease (IBD) post-treatment (Korzenik, 2019). The current study demonstrates that positive psychological interventions grounded in the PERMA model effectively enhance patient well-being, corroborating findings from previous research (Wynne et al., 2019). The PERMA model provides a thorough definition of happiness and delineates five distinct components of well-being: positive emotions, engagement, relationships, meaning and achievement. Collectively, these measurable factors contribute to an individual’s well-being and can effectively mitigate negative thinking, especially in challenging situations. Additionally, researchers continue to instill hope for recovery in patients with IBD while providing positive incentives and support for their personal attributes. This approach not only rectifies the patient’s perception of negative emotions, but also reduces the self-perceived burden associated with the disease. As a result, it alleviates the fear and anxiety associated with illness, enhances self-esteem, improves self-management, and ultimately enhances overall well-being (Schoultz et al., 2015; Gracie et al., 2017).

The residual symptoms and dysfunction associated with inflammatory bowel disease (IBD), compounded by the fear of disease recurrence, significantly impact patients’ quality of life, which is a crucial indicator of individual well-being. Previous research has explored the effects of positive psychological interventions grounded in the PERMA model on breast cancer patients, with findings affirming that this program can enhance patients’ quality of life and demonstrating promising potential for broader application (Fang et al., 2023). In the current study, the study group exhibited significantly lower post-intervention scores in the overall Fear of Progression Questionnaire-Short Form (FoP-Q-SF) compared to the control group. This improvement may be attributed to the enhanced recovery of physical function and psychological status within the study group, achieved through a multifaceted intervention approach. Specifically, social activities and physical exercise significantly facilitated recovery in physical activity, increased patients’ sense of control over their behavior, and facilitated a transition from passive to active engagement in managing their condition (Eckert et al., 2019; Kim et al., 2021). In addition, positive psychological interventions improved patient-family interactions and overall quality of life. Ultimately, these findings suggest that combining social activities, physical exercise, and positive psychological interventions may help alleviate fear of disease progression and recurrence in patients with inflammatory bowel disease (IBD), thereby enhancing their quality of life. Future research should explore the applicability and effectiveness of this combined intervention strategy in patients with IBD.

The present study is subject to several limitations that warrant consideration. Firstly, the relatively short duration of the randomized controlled trial limits our ability to sufficiently assess the long-term effects of the intervention. Therefore, future research should include longer follow-up periods to assess the sustained impact of interventions over time. Secondly, the study sample was drawn exclusively from a single tertiary hospital in Wuxi, which may limit the generalizability of the findings to a broader population. Consequently, future investigations should consider multi-center and large-sample clinical trials to enhance the robustness and external validity of the results. Thirdly, the control group in this study received standard care rather than targeted psychological interventions, which limits our ability to directly compare the efficacy of active psychological interventions with other active interventions. Thus, future studies should consider incorporating alternative interventions in the control group to provide a more rigorous assessment of the effectiveness of interventions.

## 5 Conclusion

The application of positive psychological interventions based on the PERMA model in patients with inflammatory bowel disease has effectively reduced the fear of recurrence, improved psychological resilience, and enhanced patients’ sense of well-being and overall quality of life. Future studies with larger sample sizes and extended follow-up periods are necessary to validate its long-term impact on the psychological state of patients with this disease.

## Data Availability

The raw data supporting the conclusions of this article will be made available by the authors, without undue reservation.

## References

[ref1] Barberio B. Zamani M. Black C. J. Savarino E. V. Ford A. C. (2021). Prevalence of symptoms of anxiety and depression in patients with inflammatory bowel disease: a systematic review and meta-analysis. Lancet Gastroenterol. Hepatol. 6, 359–370. doi: 10.1016/s2468-1253(21)00014-5, PMID: 33721557

[ref2] Bisgaard T. H. Allin K. H. Keefer L. Ananthakrishnan A. N. Jess T. (2022). Depression and anxiety in inflammatory bowel disease: epidemiology, mechanisms and treatment. Nat. Rev. Gastroenterol. Hepatol. 19, 717–726. doi: 10.1038/s41575-022-00634-6, PMID: 35732730

[ref3] Calviño-Suárez C. Ferreiro-Iglesias R. Bastón-Rey I. Barreiro-de Acosta M. (2021). Role of quality of life as endpoint for inflammatory bowel disease treatment. Int. J. Environ. Res. Public Health 18:7159. doi: 10.3390/ijerph18137159, PMID: 34281095 PMC8296948

[ref4] Campbell A. (1976). Subjective measures of well-being. Am. Psychol. 31, 117–124. doi: 10.1037/0003-066X.31.2.117 1267244

[ref5] Cano M. Castro F. G. De La Rosa M. Amaro H. Vega W. A. Sánchez M.. (2020). Depressive symptoms and resilience among Hispanic emerging adults: examining the moderating effects of mindfulness, distress tolerance, emotion regulation, family cohesion, and social support. Behav. Med. 46, 245–257. doi: 10.1080/08964289.2020.1712646 31935162 PMC7358125

[ref6] Chen B. Luo T. Cai Q. Pan F. Liang D. Hu Y. (2022). Effect of psychological intervention-assisted comfort nursing based on PERMA model on stress and psychological changes of patients after breast Cancer surgery. Comput. Math. Methods Med. 2022, 1–8. doi: 10.1155/2022/1853754, PMID: 35712008 PMC9197632

[ref7] Custers J. A. E. Gielissen M. F. M. Janssen S. H. V. de Wilt J. H. W. Prins J. B. (2016). Fear of cancer recurrence in colorectal cancer survivors. Support Care Cancer 24, 555–562. doi: 10.1007/s00520-015-2808-4, PMID: 26108170 PMC4689743

[ref9] Duckworth A. L. Steen T. A. Seligman M. E. (2005). Positive psychology in clinical practice. Annu. Rev. Clin. Psychol. 1, 629–651. doi: 10.1146/annurev.clinpsy.1.102803.144154, PMID: 17716102

[ref10] Eckert K. G. Abbasi-Neureither I. Köppel M. Huber G. (2019). Structured physical activity interventions as a complementary therapy for patients with inflammatory bowel disease - a scoping review and practical implications. BMC Gastroenterol. 19:115. doi: 10.1186/s12876-019-1034-9, PMID: 31266461 PMC6604412

[ref11] Eldridge S. M. Chan C. L. Campbell M. J. Bond C. M. Hopewell S. Thabane L.. (2016). CONSORT 2010 statement: extension to randomised pilot and feasibility trials. BMJ 355:5239. doi: 10.1136/bmj.i5239, PMID: 27777223 PMC5076380

[ref12] Fan X. (1999). Well-being index, overall emotion index (index of well-bing, IWB). Chin. J. Ment. Health, 82–83.

[ref13] Fang H. Zeng Y. Liu Y. Zhu C. (2023). The effect of the PERMA model-based positive psychological intervention on the quality of life of patients with breast cancer. Heliyon 9:e17251. doi: 10.1016/j.heliyon.2023.e17251, PMID: 37416631 PMC10320023

[ref14] Frangou E. Bertelli G. Love S. Mackean M. J. Glasspool R. M. Fotopoulou C.. (2021). OVPSYCH2: a randomized controlled trial of psychological support versus standard of care following chemotherapy for ovarian cancer. Gynecol. Oncol. 162, 431–439. doi: 10.1016/j.ygyno.2021.05.024, PMID: 34059348

[ref15] Gecse K. B. Vermeire S. (2018). Differential diagnosis of inflammatory bowel disease: imitations and complications. Lancet Gastroenterol. Hepatol. 3, 644–653. doi: 10.1016/s2468-1253(18)30159-6, PMID: 30102183

[ref16] Gleave A. Shah A. Tahir U. Blom J. J. Dong E. Patel A.. (2025). Using diet to treat inflammatory bowel disease: a systematic review. Am. J. Gastroenterol. 120, 83–97. doi: 10.14309/ajg.0000000000002973, PMID: 39056556

[ref17] Gracie D. J. Irvine A. J. Sood R. Mikocka-Walus A. Hamlin P. J. Ford A. C. (2017). Effect of psychological therapy on disease activity, psychological comorbidity, and quality of life in inflammatory bowel disease: a systematic review and meta-analysis. Lancet Gastroenterol. Hepatol. 2, 189–199. doi: 10.1016/s2468-1253(16)30206-0, PMID: 28404134

[ref18] Gravina A. G. Pellegrino R. Palladino G. Imperio G. Ventura A. Cipullo M.. (2025). Profiling the patient with inflammatory bowel disease in the relationship between physical activity and partner/social network status: a post hoc patient-tailored analysis of the "BE-FIT-IBD" study. Gastroenterol. Hepatol. 48:502203. doi: 10.1016/j.gastrohep.2024.502203, PMID: 38723769

[ref19] Grosvenor L. P. Errichetti C. L. Holingue C. Beasley J. B. Kalb L. G. (2023). Self-report measurement of well-being in autistic adults: psychometric properties of the PERMA profiler. Autism Adulthood 5, 401–410. doi: 10.1089/aut.2022.0049, PMID: 38116049 PMC10726181

[ref20] Guyatt G. Mitchell A. Irvine E. J. Singer J. Williams N. Goodacre R.. (1989). A new measure of health status for clinical trials in inflammatory bowel disease. Gastroenterology 96, 804–810. doi: 10.1016/0016-5085(89)90905-0 2644154

[ref21] Harvey R. F. Bradshaw J. M. (1980). A simple index of Crohn's-disease activity. Lancet 315:514. doi: 10.1016/s0140-6736(80)92767-1, PMID: 6102236

[ref22] Hodson R. (2016). Inflammatory bowel disease. Nature 540:S97. doi: 10.1038/540S97a, PMID: 28002398

[ref23] Hu P. (2012). Understanding the consensus on diagnosis and management of inflammatory bowel disease (Guangzhou, 2012). Chin. J. Gastroenterol. 17, 709–711. doi: 10.3969/j.issn.1008-7125.2012.12.002

[ref24] Jangi S. Holmer A. K. Dulai P. S. Boland B. S. Collins A. E. Pham L.. (2021). Risk of relapse in patients with ulcerative colitis with persistent endoscopic healing: a durable treatment endpoint. J. Crohns Colitis 15, 567–574. doi: 10.1093/ecco-jcc/jjaa184, PMID: 32914194 PMC8023862

[ref25] Kane S. V. Brixner D. Rubin D. T. Sewitch M. J. (2008). The challenge of compliance and persistence: focus on ulcerative colitis. J. Manag. Care Pharm. 14:s2-12; quiz s13-15. doi: 10.18553/jmcp.2008.14.s1-a.1a, PMID: 18240888 PMC10438168

[ref26] Kim Y. Carver C. S. Spillers R. L. Love-Ghaffari M. Kaw C. K. (2012). Dyadic effects of fear of recurrence on the quality of life of cancer survivors and their caregivers. Qual. Life Res. 21, 517–525. doi: 10.1007/s11136-011-9953-0, PMID: 21691928

[ref27] Kim B. Chae J. Kim E. H. Yang H. I. Cheon J. H. Kim T. I.. (2021). Physical activity and quality of life of patients with inflammatory bowel disease. Medicine (Baltimore) 100:e26290. doi: 10.1097/md.0000000000026290, PMID: 34232167 PMC8270579

[ref28] Korzenik J. (2019). Don't worry, be happy: psychological interventions in inflammatory bowel disease. Gastroenterology 156, 856–857. doi: 10.1053/j.gastro.2019.02.013, PMID: 30776343

[ref29] Le Berre C. Danese S. Peyrin-Biroulet L. (2023). Can we change the natural course of inflammatory bowel disease? Ther. Adv. Gastroenterol. 16:17562848231163118. doi: 10.1177/17562848231163118, PMID: 37153497 PMC10159495

[ref30] Luo D. (2018). The development and psychometric assessment of the strength and resilience scale for inflammatory bowel disease: [master’s thesis]. Nanjing: Nanjing Medical University.

[ref31] Lyu M. M. Siah R. C. Lam A. S. L. Cheng K. K. F. (2022). The effect of psychological interventions on fear of cancer recurrence in breast cancer survivors: a systematic review and meta-analysis. J. Adv. Nurs. 78, 3069–3082. doi: 10.1111/jan.15321, PMID: 35696315

[ref32] Mattila K. Rankala R. Voutilainen M. Mustonen A. (2022). Inflammatory bowel disease: perceived impact on leisure-time activities. Scand. J. Gastroenterol. 57, 930–935. doi: 10.1080/00365521.2022.2042593, PMID: 35196200

[ref33] Mehnert A. Herschbach P. Berg P. Henrich G. Koch U. (2006). Fear of progression in breast cancer patients--validation of the short form of the fear of progression questionnaire (FoP-Q-SF). Z. Psychosom. Med. Psychother. 52, 274–288. doi: 10.13109/zptm.2006.52.3.274, PMID: 17156600

[ref34] Mizrahi M. C. Reicher-Atir R. Levy S. Haramati S. Wengrower D. Israeli E.. (2012). Effects of guided imagery with relaxation training on anxiety and quality of life among patients with inflammatory bowel disease. Psychol. Health 27, 1463–1479. doi: 10.1080/08870446.2012.691169, PMID: 22646975

[ref35] Ng S. C. Shi H. Y. Hamidi N. Underwood F. E. Tang W. Benchimol E. I.. (2017). Worldwide incidence and prevalence of inflammatory bowel disease in the 21st century: a systematic review of population-based studies. Lancet 390, 2769–2778. doi: 10.1016/s0140-6736(17)32448-0, PMID: 29050646

[ref36] Park J. Cheon J. H. (2021). Incidence and prevalence of inflammatory bowel disease across Asia. Yonsei Med. J. 62, 99–108. doi: 10.3349/ymj.2021.62.2.99, PMID: 33527789 PMC7859683

[ref37] Paulides E. Cornelissen D. de Vries A. C. van der Woude C. J. (2022). Inflammatory bowel disease negatively impacts household and family life. Frontline Gastroenterol. 13, 402–408. doi: 10.1136/flgastro-2021-102027, PMID: 36046490 PMC9380757

[ref38] Rivière P. Vermeire S. Irles-Depe M. Van Assche G. Rutgeerts P. Denost Q.. (2021). Rates of postoperative recurrence of Crohn's disease and effects of immunosuppressive and biologic therapies. Clin. Gastroenterol. Hepatol. 19, 713–720.e1. doi: 10.1016/j.cgh.2020.03.064, PMID: 32272248

[ref39] Sabir G. Abdelhady H. A. Oumar Abakar A. Gangavarapu R. R. Mahmud S. A. Manandhar A.. (2024). The potential benefits of exercise in managing inflammatory bowel disease: a systematic review. Cureus 16:e68948. doi: 10.7759/cureus.68948, PMID: 39381484 PMC11461038

[ref40] Schoultz M. Atherton I. Watson A. (2015). Mindfulness-based cognitive therapy for inflammatory bowel disease patients: findings from an exploratory pilot randomised controlled trial. Trials 16:379. doi: 10.1186/s13063-015-0909-5, PMID: 26303912 PMC4549082

[ref41] Sehgal P. Ungaro R. C. Foltz C. Iacoviello B. Dubinsky M. C. Keefer L. (2021). High levels of psychological resilience associated with less disease activity, better quality of life, and fewer surgeries in inflammatory bowel disease. Inflamm. Bowel Dis. 27, 791–796. doi: 10.1093/ibd/izaa196, PMID: 32696966 PMC8128407

[ref42] Simard S. Thewes B. Humphris G. Dixon M. Hayden C. Mireskandari S.. (2013). Fear of cancer recurrence in adult cancer survivors: a systematic review of quantitative studies. J. Cancer Surviv. 7, 300–322. doi: 10.1007/s11764-013-0272-z, PMID: 23475398

[ref43] Sirois F. M. Hirsch J. K. (2017). A longitudinal study of the profiles of psychological thriving, resilience, and loss in people with inflammatory bowel disease. Br. J. Health Psychol. 22, 920–939. doi: 10.1111/bjhp.12262, PMID: 28804983

[ref44] Tiles-Sar N. Neuser J. de Sordi D. Rücker G. Baltes A. Preiss J.. (2022). Psychological interventions for inflammatory bowel disease: a systematic review and component network meta-analysis protocol. BMJ Open 12:e056982. doi: 10.1136/bmjopen-2021-056982, PMID: 35732389 PMC9226957

[ref45] Valibouze C. Desreumaux P. Zerbib P. (2021). Post-surgical recurrence of Crohn's disease: situational analysis and future prospects. J. Visc. Surg. 158, 401–410. doi: 10.1016/j.jviscsurg.2021.03.012, PMID: 33858790

[ref46] von Wietersheim J. Köhler T. Feiereis H. (1992). Relapse-precipitating life events and feelings in patients with inflammatory bowel disease. Psychother. Psychosom. 58, 103–112. doi: 10.1159/000288617, PMID: 1484919

[ref8] Walmsley R. S. Ayres R. C. Pounder R. E. Allan R. N. (1998). A simple clinical colitis activity index. Gut 43:29–32. doi: 10.1136/gut.43.1.29, PMID: 9771402 PMC1727189

[ref47] Wu Q. Ye Z. Li L. Liu P. (2015). Reliability and validity of Chinese version of fear of progression questionnaire-short form for cancer patients. Chin. J. Nurs. 50, 1515–1519. doi: 10.3761/j.issn.0254-1769.2015.12.021

[ref48] Wynne B. McHugh L. Gao W. Keegan D. Byrne K. Rowan C.. (2019). Acceptance and commitment therapy reduces psychological stress in patients with inflammatory bowel diseases. Gastroenterology 156, 935–945.e1. doi: 10.1053/j.gastro.2018.11.030, PMID: 30452919

[ref49] Yeh H. P. Stone J. A. Churchill S. M. Wheat J. S. Brymer E. Davids K. (2016). Physical, psychological and emotional benefits of green physical activity: an ecological dynamics perspective. Sports Med. 46, 947–953. doi: 10.1007/s40279-015-0374-z, PMID: 26330207

[ref50] Yu Q. Zhu C. Feng S. Xu L. Hu S. Chen H.. (2021). Economic burden and health care access for patients with inflammatory bowel diseases in China: web-based survey study. J. Med. Internet Res. 23:e20629. doi: 10.2196/20629, PMID: 33399540 PMC7815453

[ref51] Zhang J. Xu R. Wang B. Wang J. (2016). Effects of mindfulness-based therapy for patients with breast cancer: a systematic review and meta-analysis. Complement. Ther. Med. 26, 1–10. doi: 10.1016/j.ctim.2016.02.012, PMID: 27261975

[ref52] Zhou L. Lu X. (2004). Health-related quality of life in patients with inflammatory bowel disease. Chin. J. Intern. Med. 05, 76–78. doi: 10.3760/j.issn:0578-1426.2004.05.030

